# Comparative Blood Profiling Based on ATR-FTIR Spectroscopy and Chemometrics for Differential Diagnosis of Patients with Amyotrophic Lateral Sclerosis—Pilot Study

**DOI:** 10.3390/bios14110526

**Published:** 2024-10-30

**Authors:** Kateryna Tkachenko, José M. González-Saíz, Ana C. Calvo, Christian Lunetta, Rosario Osta, Consuelo Pizarro

**Affiliations:** 1 Department of Chemistry, University of La Rioja, Logroño, 26006 La Rioja, Spain; kateryna.tkachenko@unirioja.es (K.T.); josemaria.gonzalez@unirioja.es (J.M.G.-S.); 2 LAGENBIO, Centro de Investigación Biomédica en Red de Enfermedades Neurodegenerativas (CIBERNED), Aragon Institute for Health Research (IIS Aragon), Zaragoza University, Calle Miguel Servet 13, 50013 Zaragoza, Spain; accalvo@unizar.es (A.C.C.); osta@unizar.es (R.O.); 3 Department of Neurological Rehabilitation, Istituti Clinici Scientifici Maugeri IRCCS, Institute of Milan, 20138 Milan, Italy; christian.lunetta@icsmaugeri.it

**Keywords:** amyotrophic lateral sclerosis, infrared spectroscopy, metabolic signatures, patient stratification, chemometrics, classification strategy, health and wellbeing monitoring

## Abstract

Amyotrophic lateral sclerosis (ALS) is a motor neurodegenerative disease characterized by poor prognosis. Currently, screening and diagnostic methods for ALS remain challenging, often leading to diagnosis at an advanced stage of the disease. This delay hinders the timely initiation of therapy, negatively impacting patient well-being. Additionally, misdiagnosis with other neurodegenerative disorders that present similar profiles often occurs. Therefore, there is an urgent need for a cost-effective, rapid, and user-friendly tool capable of predicting ALS onset. In this pilot study, we demonstrate that infrared spectroscopy, coupled with chemometric analysis, can effectively identify and predict disease profiles from blood samples drawn from ALS patients. The selected predictive spectral markers, which are used in various discriminant models, achieved an AUROC sensitivity of almost 80% for distinguishing ALS patients from controls. Furthermore, the differentiation of ALS at both the initial and advanced stages from other neurodegenerative disorders showed even higher AUROC values, with sensitivities of 87% (AUROC: 0.70–0.97). These findings highlight the elevated potential of ATR-FTIR spectroscopy for routine clinical screening and early diagnosis of ALS.

## 1. Introduction

Amyotrophic lateral sclerosis (ALS) is a motor neurodegenerative disease primarily affecting the upper and lower motor neurons [1]. ALS is a rapidly progressive and severely debilitating condition, making prompt diagnosis and optimal therapy selection crucial for successful treatment and patient well-being. Currently, ALS diagnosis relies on clinical symptoms and electroneuromyography studies [2]. The arrangement of symptoms displayed by patients during the course of the disease reflects the progressive loss of motor neurons. Thus, patients with ALS share some overlapping features with other commonly known neurodegenerative disorders such as Parkinson’s, Alzheimer, or other neuro-disorders (ON), namely, muscular dystrophies such as Myotonic Dystrophy or Becker’s Muscular Dystrophy [3,4,5,6,7,8]. This issue often leads to delayed diagnosis and frequent misdiagnosis, making a patient’s diagnosis a challenging and time-consuming task. The survival rate for ALS patients within five years of diagnosis is alarmingly low [9]. Therefore, individuals who receive timely diagnosis and treatment exhibit more favorable outcomes [10].

Early ALS diagnosis and treatment depends on identifying specific biomarkers for ALS detection and developing reliable, user-friendly diagnostic techniques. Metabolomics studies have made significant progress in ALS research, enabling the discovery of various compounds potentially involved in the disease. For instance, differential studies between healthy individuals and ALS patients have identified lipid metabolite imbalances such as the sphingolipids involved in autophagy and inflammation [11]. Increased levels of biofluid lipids, including cholesteryl esters, triglycerides, and lipoproteins, have been associated with a higher incidence of ALS [12]. Moreover, several proteins, such as SOD1 and TDP-43, have been implicated in both familial and sporadic ALS, with genetic mutations encoding these proteins linked to altered biological pathways [13]. For example, patients with *SOD1* mutations show decreased levels of serine, lysine, and arginine, while patients with *TARDBP* mutations encoding TDP-43 exhibit increased levels of pyruvate and fatty acids. Despite extensive research, specific fluid biomarkers, including plasma and cerebrospinal fluid (CSF), are not yet available in a clinical setting. This has led us to analyze the stratification ability of vibrational spectroscopy as a diagnostic method, which uses a small amount of blood.

Fourier transform infrared (FTIR) spectroscopy emerges as a promising method in metabolomic studies [14], which is characterized by its simplicity, reproducibility, and non-destructive nature. Samples can be analyzed by FTIR spectroscopy in two principal modes: transmission and reflection. The reflection mode is widely used to acquire infrared spectra in a non-destructive way. Thus, the most used internal reflection is attenuated total reflection (ATR) because of its key advantage, namely, minimal sample preparation is required, involving only small amounts of biofluids [15]. FTIR spectroscopy provides valuable molecular-level insights, enabling the study of functional groups, bonding types, and molecular conformations. The unique spectral bands observed in vibrational spectra offer direct information about the biochemical composition of each molecule. The utility of this analytical platform has been demonstrated in different organ pathologies [16] and in a wide range of cancers [17,18] such as breast cancer [19,20], liver cancer [21,22], prostate cancer [22], and even ovarian cancer [23]. In addition, the potential of the ATR technique is often reinforced by the accuracy of results. Thus, Bury et al. [24] performed a study in which ATR spectroscopy analyzed various brain tumors from blood plasma, achieving up to 100% accuracy for high-grade glioma vs. low-grade glioma and 88–100% accuracy for meningiomas.

Attention is now being focused on serum and plasma as informative biomaterials for the low-invasive diagnosis and monitoring of systemic diseases. Blood is a complex and dynamic biological fluid that reflects alterations in the levels of many substances, such as lipids, sugars, and proteins, which can serve as indicators of systemic diseases or the initiation of new ones [25]. Thus, blood could be an effective source for screening or monitoring ALS. However, the use of IR spectroscopy of blood for ALS diagnosis is the branch that remains uncovered and must be elucidated. In the present work, ATR-FTIR spectroscopy of supernatant blood is employed to discriminate between recently diagnosed ALS patients and controls by using chemometric techniques. To the best of our knowledge, this is the first study applying the ATR-FTIR technique to the blood samples of ALS patients. Previously, interesting results were achieved by analyzing other biological samples such as the tears and tissues of ALS patients [26,27]. Nevertheless, very little importance was placed in distinguishing between ALS and similar conditions; the distinction is crucial to avoid misdiagnosis. Thus, this is also the first study that performed a differential analysis of patients with motor neuron diseases, which share similar features with ALS.

This holistic approach aims to identify differential signatures, primarily for finding early signs indicative of ALS onset, and to effectively stratify patients based on disease conditions. Our second aim was directed toward discriminating between ALS-affected patients at initial stages from those at the advanced stage of the disease and from patients characterized by other neuro states.

## 2. Materials and Methods

### 2.1. Study Population

This study included participants recruited from Niguarda Ca’Granda Hospital in Milan (Italy). Written informed consent was obtained from all patients prior to inclusion in the study to publication of their case details, which has been conducted according to the Declaration of Helsinki principles and according to the Directive 2004/23/EC of the European Parliament and of the Council and to the institutional ethical committees of Niguarda Ca’Granda Hospital (approval N·636-122015; 23 December 2015). Thus, a cohort of 76 blood samples were matched for age and gender whenever possible. Samples were divided into 35 ALS patients (characterized by familial cases due to quadruple mutation in the ALS susceptibility genes SOD1/TDP43/FUS/c9orf72, 15 controls, and 7 patients with other neuropathies (ON). The ALS group was divided into two subgroups: 19 patients were collected at first diagnosis of ALS (T0), and 16 were obtained after six months of diagnosis/treatment of ALS (T6). Regarding the ON group, only samples of 7 participants were at our disposal, and the participants were affected by Becker’s Muscular Dystrophy, Extrapyramidal syndrome, Facioscapulohumeral Muscular Dystrophy, and Myotonic Dystrophy. Any additional information, such as age, gender, or medication, was included in this study. All participants were identified by number, not by name. The study was also approved by the Ethics Committee (Comité de Etica de la Investigación de la Comunidad de Aragón or CEICA) (CP-CI PI18/078).

### 2.2. Sample Collection

Once drawn, the samples analyzed in this study were obtained after first centrifuging each Pax tube containing the blood sample for 10 min at 3000–5000 rpm and incubated for 2 h at room temperature, following the recommendations of the commercial kit (Preanalytix.Com: PAXgene—Specimen Collection & Processing, n.d., Hombrechtikon, Switzerland). The supernatant samples recovered from this first centrifugation were used to perform the analysis in this study. In addition, these samples were preserved at −80 °C for further use.

### 2.3. ATR-FTIR Measurements

Spectra were obtained using Spectrum Two FT-IR spectrometers (PerkinElmer, Waltham, MA, USA) equipped with ATR single reflection diamond and recorded in the region 1500–1000 cm^−1^ spectral range with a resolution of 4 cm^−1^. Each sample was analyzed by deposing it on an ATR diamond crystal, measured three times in 16 scans, and then averaged. After each analysis, the blood plasma spot on the ATR diamond crystal was cleaned with the cleaning tissues pre-wetted with deionized water. The temperature was maintained at 23.0 °C ± 1.0 °C while recording the signals. The Spectrum 10 software package facilitated data collection, processing, and results generation. To monitor the reproducibility and repeatability of the analysis, a fresh background absorption spectrum was recorded each time for correction.

### 2.4. Data Analysis

Given the high dimensionality of biological spectral data, various disturbing factors such as random noise, baseline distortions, and light scattering can influence spectral data acquisition and, thus, should be corrected. In addition, to compensate for instrumental artifacts and sample-to-sample variations, different preprocessing methods were evaluated individually or in combination to minimize adulterant-unrelated variability. Thus, normalization and moving average combined with Savitzky–Golay (S–G) second derivatives (11 points) were applied to ATR spectra. These steps ensured better resolution of overlapping peaks and decreased scatter effects. The original and preprocessed spectra are provided in Supplementary Figures S1 and S2.

The test set was selected randomly for each classification step. Thus, in each classification approach, a training set and a test set were used to develop and validate the classification proposed. Corrected and normalized output matrices were auto scaled before additional multivariate analysis.

Raw metabolic data were processed and analyzed using Unscrambler (version X 11.0, Camo ASA, Oslo, Norway), V-Parvus (version PARVUS2011, Michele Forina, Genoa, Italy), and MATLAB (version 9.13.0 (R2022b), Natick, MA, USA). Based on previous knowledge and experience regarding higher spectral regions, which are prone to noise, water absorption, and saturation, only the mid-IR spectrum was analyzed. Specifically, the biochemical “fingerprint region” at 1500–1000 cm^−1^ was examined, reducing notably analytical time.

Firstly, Principal Component Analysis (PCA) was performed for the initial data overview and to remove possible outliers. Therefore, Linear Discriminant Analysis (LDA) [28], a widely used and robust supervised chemometric classification technique, was employed to differentiate between predefined subgroups. LDA ensures that each object is assigned to one of the specified classes based on established classification rules. To enhance the discrimination performance, the most significant wavenumbers were selected through stepwise orthogonalization of predictors using the SELECT algorithm [29]. This approach allowed for reliable classification of patient classes and provided key features for subsequent classification analysis. To reduce the risk of overfitting during LDA-based variable selection, careful attention must be given to the number of variables used in building the classification model. It is recommended that the number of training samples be at least three times higher than the number of variables retained by the SELECT process. Additionally, internal cross-validation was utilized to evaluate the predictive discrimination of each classification model. This involved selecting and decorrelating key variables based on their maximum correlation weight, a vital approach when dealing with high-dimensional datasets. By filtering out non-essential features and focusing on significant variables, the analysis was refined for subsequent steps. Cross-validation (CV) was employed to fine-tune the classifications, while external validation assessed the accuracy of predictions. The effectiveness of the resulting discriminant rules was measured against several important criteria.

Next, Partial Least Squares Discriminant Analysis (PLS-DA) and Orthogonal Projections to Latent Structures Discriminant Analysis (OPLS-DA) were performed to minimize potential contradictions within groups and enhance the differentiation between samples. The variable loadings from a validated OPLS-DA model were used to rank variables based on their effectiveness in distinguishing between groups. A 5-fold internal cross-validation was applied, and Q2Y (predictive ability estimated through cross-validation) and R2Y (goodness of fit) values were extracted to assess the quality of the resulting models. CV-ANOVA and permutation tests were also employed to further validate the models.

To explore the differences between sample groups, the loading plots and Variable Importance in Projection (VIP) scores from each model were carefully analyzed. These analyses helped identify key variables that contribute to group discrimination. Receiver Operating Characteristic (ROC) curves, along with Area Under the Curve (AUC) values, were plotted to evaluate the diagnostic performance of selected infrared bands for distinguishing between control individuals and diseased patients.

## 3. Results

The spectral region that allowed for the discrimination between different patient groups was located in the ‘’fingerprint region’’, which comprises a number of bands that characterize different biochemical compounds, such as amino acids, lipids and phospholipids, and carbohydrates.

Thus, the region at 1450–1400 cm^−1^ is characterized by asymmetric and symmetric methyl bending modes. Meanwhile, several spectral regions are attributable to absorption bands of lipids: the region of 1500–1350 cm^−1^ belonging to –CH_2_ and –CH_3_ vibrations from the lipid acyl chains and the region of 1270–1000 cm^−1^ for asymmetric and symmetric vibrations of –PO^2−^ in phospholipid. Meanwhile, stretching vibrations of the C–O/C–C groups belong to bands located between 1200 and 800 cm^−1^, and deformational modes of the CH_3_/CH_2_ of carbohydrate spectra are assigned to 1500–1200 cm^−1^ bands [30]. The spectra of nucleic acids are attributed to different spectral regions: from 1550 to 1270 cm^−1^ for the deformation vibrations of the bases coupled with the sugar vibrations, the region of 1270–1000 cm^−1^ for vibrations of –PO^2−,^ or sugar-phosphate backbone vibrations.

### 3.1. Discrimination Between ALS (T0) and Controls

**Figure 1:**
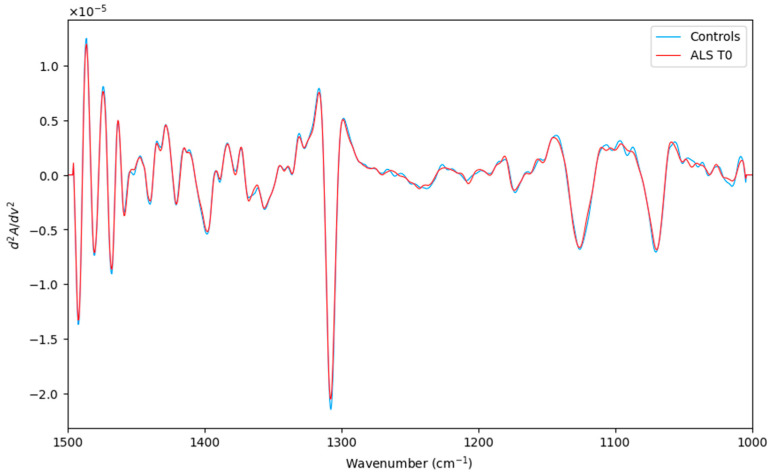
Averaged second derivative spectra of the corresponding ALS (T0) category (red line) and Controls (green line) in the spectral region (1500–1000 cm^−1^).

After careful preprocessing (Figure 1), ATR-FTIR measurements were submitted for further multivariate analysis. In this preliminary analysis, the principal goal was to evaluate the prognostic ability of the ATR-FTIR technique. Thus, a pairwise comparison, including ALS (T0) and healthy controls (a total of 35 objects), was studied.

#### 3.1.1. SELECT-LDA Strategy

As good data analysis practice, unsupervised PCA was performed to identify possible clusters and outliers. Therefore, simultaneous feature selection and classification were performed by applying a stepwise decorrelation procedure (SELECT). Since it allows for the optimization of discrimination by avoiding redundant information in the subset of selected spectral variables, it is usually prioritized among other variable selection techniques.

**Figure 2:**
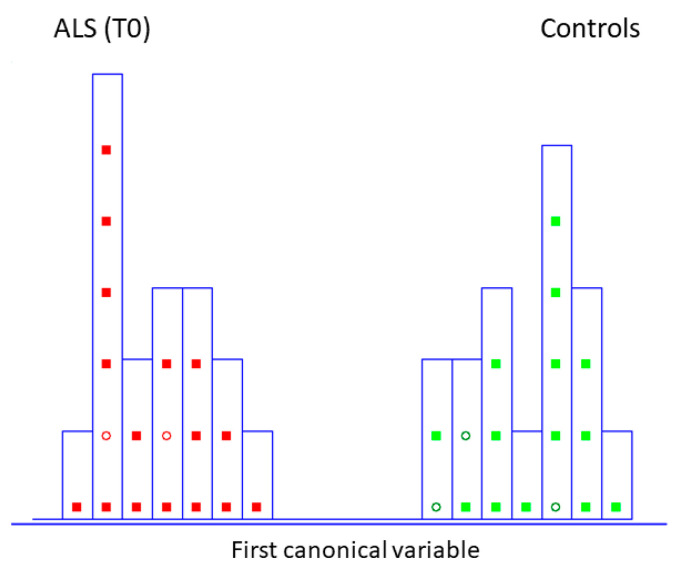
Histogram of the first canonical variable for the discrimination of ALS patients at the initial stage (T0) (■) and healthy controls at the same time of blood collection (T0) (■) after performing SELECT-LDA (*y*-axis indicates the maximum discrimination power between categories). Test samples are displayed as circles (◯).

This chemometric approach has already been widely applied, which extracts the most significant wavenumbers from the IR dataset and provides input features for further classification and class modeling strategies for the discrimination of pathological status [31]. A careful reading of the commonly established rule to understand finally selected wavenumbers is encouraged [32]. Thus, ten spectra variables (the number which is almost three times smaller than the number of training objects present in the data matrix) were decorrelated from other variables by SELECT algorithm (1400.5, 1335.5, 1180, 1033.5, 1475, 1355, 1234, 1449.5, 1310.5, 1185 cm^−1)^. LDA, built by leave one out (LOO) cross-validation, was performed to evaluate the feasibility of this classification methodology to differentiate between patients characterized by recently diagnosed ALS and the control group. Excellent discrimination among categories was achieved, providing a 100% level of correctly classified samples for diseased subjects and controls. In addition, excellent external prediction performances of 100% were achieved using five patients in the external test set for both categories (within no misclassified patients), respectively (Table 1). Furthermore, discriminative histogram (Figure 2) represents a clear interclass separation achieved between two categories. As can be observed, ALS patients exhibit a metabolic profile so similar that they are perfectly separated and segregated from the controls; any patient is detached from their class.

#### 3.1.2. SIMCA Class Modeling Strategy

The subset of ten IR signatures selected by the SELECT algorithm was used to build optimized class models based on SIMCA [33]. In other classification methods, a new sample will always be classified in one of the predefined categories based on the development of classification rules and delimiters between classes. Whereas in class models, significance limits are built for the specified classes [30]; for this reason, SIMCA often outperforms other classification strategies.

Herein, SIMCA modeling was performed on previously selected spectral variables for the classification problem ALS (T0) vs. Controls. A class modeling of ten variables achieved satisfactory results in both internal (LOO) 83.33% and external predictions 100.00%, respectively (Table 2) and (Figure S3). Considering this is a pilot study, the internal prediction showed lower accuracy due to a reduced sample number. These results should be cautiously retested utilizing a more significant number of samples, though the results are still promising. Likewise, to model ALS (T0) vs. controls, the same number of external predictors were used for LDA classification, and an equally high external prediction was achieved. Meanwhile, the values of the discriminant power (DP) and modeling power (MP) are summarized in Table 3.

The MP describes how well a variable helps each principal component model variation in the data, and all values are close to 1. Meanwhile, DP describes how well a variable helps each principal component model classify samples in a training set. Thus, the selected variable for this class modeling showed very high values of discriminant power, indicating the contribution of each one in class separation.

#### 3.1.3. OPLS-DA Strategy

Additionally, Orthogonal Projections to Latent Structures Discriminant Analysis (OPLS-DA), another supervised classificatory technique, was used in this study. This strategy is favorable in biomedical spectroscopy to decrease systematic variation due to experimental bias or biological variation [34].

Thus, OPLS-DA tends to produce less complex discriminant models by identifying a more refined multivariate subspace for maximum group separations by applying [35]. Thus, OPLS-DA enables group separations thanks to its capability to distinguish subtle variations in MIR datasets based on spectral features.

**Figure 3:**
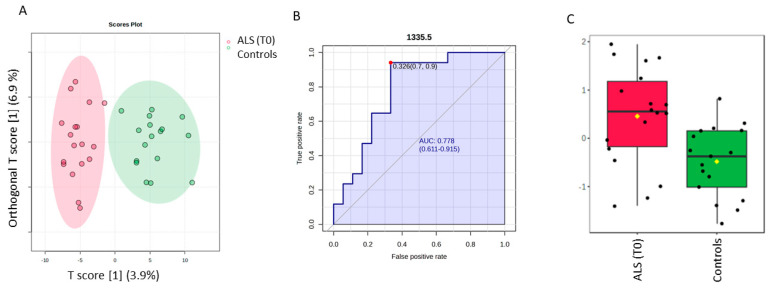
Potential of the wavenumber 1335.5 cm^−1^ to differentiate between the spectra of the serum samples from the control group and ALS (T0) group. (**A**) OPLS-DA analysis between ALS T0 (red T2 Hotelling’s ellipses with a 95% confidence level) and healthy controls (green T2 Hotelling’s ellipses with a 95% confidence level). (**B**) Receiver operating characteristic (ROC) curve is developed while the wavenumber 1335.5 cm^−1^ is considered, with its respective parameters, including the area under the curve (AUC); the dot represents the selected cut point, defined based on the Youden index (J). (**C**) box plot classification of samples from the control group and from the ALS group.

The discrimination between ALS (T0) and controls by a supervised OPLS-DA analysis is highlighted in Figure 3, whereas the permutation test with one predictive and one orthogonal component revealed a statistical discriminant model that was not so high (Figure S4). Nevertheless, our aim was to investigate the ten most discriminant spectral variables based on the Variable in projection score (VIP ≥ 1), which contributed to the model, to make a comparative analysis with those obtained for the SELECT-LDA classification. Thus, one interesting spectra value was identified. The spectral band at 1335.5 cm^−1^ was also selected among the ten that were the most important by performing SELECT-LDA classification. The predictive ability of this wavenumber with *p* < 0.05 was evaluated using the ROC curve. This curve is a graphical plot of the sensitivity versus the (1-) specificity, determining several possible cut points for the test (a cut point that maximizes the accuracy of the diagnostic test [36]. Thus, the wavenumber 1335.5 cm^−1^ selected by both classification strategies showed an AUC of 0.778 and *p* < 0.0038. The optimal cut point was defined at ≤0.326, resulting in a sensitivity of 90% and specificity close to 70%, indicating that it has good diagnostic potential for patients with amyotrophic lateral sclerosis. Selected spectral wavenumbers by each classification model are summarized in Table S1.

### 3.2. Discrimination Between ALS (T0), ALS (T6), and ON

#### 3.2.1. SELECT-LDA Classification

**Figure 4:**
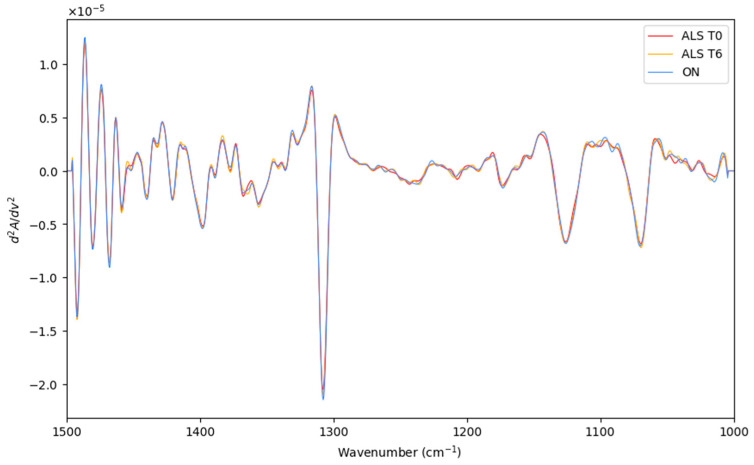
The preprocessed and averaged spectra of recently diagnosed ALS patients (T0), patients after 6 months of diagnosis ALS (T6), and patients with other neurodegenerative motor pathologies (ON).

Herein, the LDA classification of the recorded spectra of recently diagnosed ALS patients (T0), patients after 6 months of diagnosis ALS (T6), and patients with other neurodegenerative motor pathologies (ON) were performed. The preprocessed and averaged spectra of each category are represented in Figure 4. Before LDA analysis, as explained above, SELECT was applied to extract those predictor variables correlated with the discrimination between the categories considered here. Therefore, based on the SELECT rules, ten variables (1393.5, 1304, 1231.5, 1106, 1150.5, 1135.5, 1319, 1045, 1341.5, and 1449.5) were decorrelated from other signals and used for LDA analysis. Interestingly, in this classification approach, the spectral band at 1449.5 cm^−1^ was also selected as important, indicating that it can be characteristic of the ALS T0 category.

**Figure 5:**
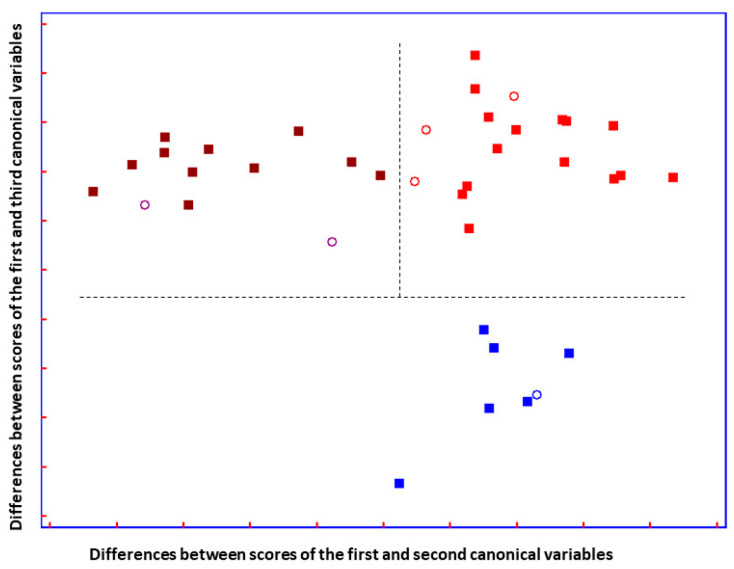
Plot of the differences between discriminant scores for supernatant blood samples after performing SELECT-LDA to discriminate between three groups of samples: ALS T0 (■), ALS T6 (■), and ON (■) subjects. Test samples are displayed as unfilled circles (◯). The model was performed using the ten decorrelated wavenumbers by SELECT.

The results of SELECT-LDA performance are displayed in Table 4. The goodness of the classification strategy performed on ten selected IR spectral signatures can also be visually appreciated in Figure 5.

The total LDA classification ability yielded 98.73%; meanwhile, the overall prediction ability of the model showed 87.50%. Compared to previous optimal separation performed to distinguish patients based on the advancement of the disease, herein, among 14 ALS (T0) patients, one subject was misclassified as patients with advanced ALS (T6) and one as having other motor neuron disease. (Table S1). In addition, two out of eleven ALS (T6) patients were classified as recently diagnosed patients with ALS. These results could reflect some issues due to the heterogeneity of ALS phenotype and confirmed that the progression of the disease was not so pronounced, in terms of metabolic evolution, as it was previously reported [37]. Nevertheless, a test set of six samples not included in the training set showed perfect prediction ability, and all patients were perfectly classified.

A good group separation can be appreciated in Figure 5. The three groups of patients affected by similar neurodegenerative disturbances are perfectly separated. Interestingly, it can be observed that the two ALS disease-carrying groups are almost not separated on the *y*-axis, but they are separated on the *x*-axis. Additionally, imaginable delimiters highlight the fact that ALS groups are greatly distinguished from patients carrying other neuro disorders, suggesting that ALS groups are characterized by changes in metabolic profile that do not characterize patients with ON.

#### 3.2.2. PLS-DA Analysis

**Figure 6:**
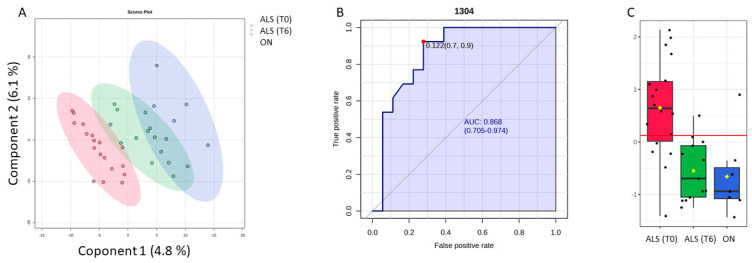
Potential of the wavenumber 1304 cm^−1^ to differentiate between the spectra of the blood samples from the ALS (T0), ALS (T6), and ON groups. (**A**) PLS-DA discriminative analysis between ALS T0 (red T2 Hotelling’s ellipses with a 95% confidence level), ALS (T6) (green T2 Hotelling’s ellipses with a 95% confidence level), and ON group (blue T2 Hotelling’s ellipses with a 95% confidence level). (**B**) Receiver operating characteristic (ROC) curve developed considering the wavenumber 1304 cm^−1^, with its respective parameters, including the area under the curve (AUC); the dot represents the selected cut point, defined based on the Youden index (J). (**C**) Box plot classification of samples from both ALS groups (T0) and (T6) and ON group.

Additionally, the PLS-DA approach was also performed as a variable selection method. It can be observed that all three patient groups are perfectly segregated from each other (Figure 6). It seems that some overlap is present between the ALS (T6) and ON categories, but on the PLS-DA 3D score plot, it is observable that the clusters are not overlapped (see Figure S5). The parameters used to evaluate the performance of the model were: R2 is the coefficient of determination, which measures the proportion of variance in the dependent variable that is predictable from the independent variables; and Q2 is the predictive squared correlation coefficient, which measures the predictive ability of the model based on cross-validation and accuracy (see Supplementary Materials).

To make the selected variables comparable by two classification methods, only 10 spectra variables were selected based on the VIP score (≥1). Thus, among the selected spectral bands by the PLS-DA model, the band 1304 cm^−1^, which was previously decorrelated from other variables to perform SELECT-LDA classification, was also significant in this model. Thus, the predictive ability of this wavenumber with *p* < 0.05 was evaluated using the ROC curve. Therefore, the wavenumber 1304 cm^−1^ selected by both chemometric classification strategies had an AUC of 0.868 and *p* < 0.00003. The optimal cut point was defined at ≤0.122, resulting in a sensitivity of 92% and a specificity close to 70%, indicating that it has a good potential for the discrimination of different stages of ALS disease and its differentiation from other motor neuron diseases.

## 4. Discussion

In this pilot study, the relevance of ATR-FTIR spectroscopy for the diagnosis of ALS disease has been explored. The metabolic fingerprint acquired from MIR spectra allows for the identification of the broad biochemical alterations induced by the underlying disease. In order to perform spectral analysis, chemometric techniques were applied to reduce the number of spectral variables from a thousand to approximately a dozen in order to minimize the influence of noisy and/or redundant variables. The principal aim of this study was to evaluate the ability of MIR-spectra to obtain a global metabolic/spectroscopic profile useful for the assessment of short-term prognosis of patients with amyotrophic lateral sclerosis. Thus, the most discriminant wavenumbers based on different chemometric classification strategies were evaluated. We investigated if it is possible to segregate directly ALS patients at the initial stage from healthy controls and if this method is sensitive to discriminate between ALS at different progression stages and other motor neuron diseases. Therefore, some wavenumbers became significant after different classification approaches had been applied. Thus, to identify the most discriminative spectra biomarkers to distinguish early diagnosed ALS patients from healthy controls, ten spectral features (1400.5, 1335.5, 1180, 1033.5, 1475, 1355, 1234, 1449.5, 1310.5, 1185 cm^−1^) were obtained through the SELECT algorithm, achieving a classification between ALS at the initial stage and healthy controls with 100% sensitivity, 100% specificity, and 100% overall accuracy.

In fact, the most useful ability above all is to prevent neurodegeneration and start the treatment as soon as possible.

The designed chemometric strategy to distinguish contemporary ALS disease progression and other neuro disorders, which is important in second place for patient well-being and can avoid misdiagnosis, also exhibited very good classification accuracy (~98%), identifying the most important spectral biomarkers that majorly contribute to patients’ variability. Our method based on different classification steps showed excellent discriminative results and pretty good prediction accuracy (~85%) for this classification. We can speculate that the two patients in the ALS category (T6) were misclassified as those with recently diagnosed ALS because their metabolic profile has not yet undergone the changes typically characterizing an advanced stage of ALS. Moreover, it should be considered that since these patients received the diagnosis, it is more likely that they started treatment; thus, in these specific patients, the treatment response could contribute to decelerating the progression of ALS. This possible explanation to justify misclassified ALS patients actually confirms everything that has been said so far, that the sooner the disease is diagnosed, the sooner the treatment begins, improving the patient’s well-being. In addition, another plausible reason regarding the different ALS genotypes could just be that these two subjects belong to different disease phenotypes, and therefore, based on their genotype, they can manifest a slower progression of ALS. In addition, two patients with recently diagnosed ALS were misclassified as those belonging to ALS (T6) and ON categories. Maybe this misclassification is due to some similarities in patients’ metabolic profiles, which characterize neurodegeneration in the central nervous system. Thus, these findings are also useful for the problem, and the exhaustive collection of information completing the patient’s dataset would confirm ATR-FTIR’s ability to predict possible disease initiation. This speculation could be the basis for further interesting investigation to discriminate between patients with different genetic backgrounds or patients on treatment versus patients without treatment.

The development of medical diagnostics using vibrational spectroscopy involves two main steps: the classification of spectral biomarkers and the biochemical assignment of these identified biomarkers, respectively. In this context, we explore the potential role of the bands that were consistently selected across different classification strategies.

Both predictive spectra wavenumbers, 1304 and 1335.5 cm^−1^, identified by diverse classification strategies belong to the region characteristic of vibrational changes in different chemical bonds in Amide III-band and Proteins (~1350–1220) [38]. Interestingly, Martel et al. [39], by performing the FTIR technique to differentiate between ALS-derived tissue-engineered skins and controls, also identified this spectral region as important and discriminative for ALS diagnosis.

Additionally, the spectral band at 1449 cm^−1^ emerged as significant in both classification models, which exclusively included the ALS (T0) category. This suggests that this band may be a specific marker for this group. The 1449 cm^−1^ band is likely associated with the methylene deformation or methyl bending of lipids, indicating alterations in lipid metabolism. Interestingly, in our previous study on Parkinson’s disease (PD), certain bands within this spectral region also distinguished PD from controls. This parallel hints at a common underlying mechanism, possibly related to oxidative stress in the central nervous system, which is characterized by increased lipid peroxidation, elevated superoxide dismutase activity, and altered zinc levels [40,41]. Such changes in the metabolic profile of ALS patients are consistent with the known pathophysiology of the disease. Our findings suggest that ATR-FTIR spectroscopy combined with chemometric analysis can be potentially employed for rapid and accurate screening and diagnosis of ALS disease.

## 5. Conclusions

ATR-FTIR spectroscopy is an emerging platform used to detect spectral biomarkers in different malignancies and disorders. It allows the delivery of rapid information, which is particularly useful in cases when a result is urgently required. Considering that amyotrophic lateral sclerosis has a very high mortality rate, the correct identification and early diagnosis would significantly improve success in this field, enabling early-stage therapeutic intervention. In this pilot study, the ATR-FTIR analysis coupled with chemometric techniques showed very high prediction accuracy, which allows for patient screening and differentiation. In addition, some predictive spectra were identified by different algorithms, which provide a good predictive ability to distinguish between ALS and healthy subjects and between ALS at different stages of the disease and other neuro disorders, which is extremely important for avoiding misdiagnosis. This analytical method does not purport to resolve the question of final diagnosis. Still, it could be helpful for screening blood samples of multiple disorders by providing spectral biomarkers for the initial objective diagnosis or monitoring of disease progression. However, further evaluation of specificity is necessary, particularly through studies involving a large cohort of patients with diseases that share common features with ALS. Therefore, the correlation of spectroscopic data with molecular markers, which are derived from multi-omics approaches, is encouraged.

## Figures and Tables

**Table 1:** Results of SELECT-LDA performance to discriminate between HC and ALS at the initial stage in both classification and internal/external validations.

Group	Classification (%)	Prediction (%)	External Prediction (%)	Total Rate (%)
ALS (T0)	100	100	100	100
HC	100	100	100	100
Total rate	100	100	100	100

**Table 2:** The results of SIMCA class-modeling performance on ten IR-selected spectra variables to discriminate between patients with ALS onset and controls.

10 Variables	Classification (%)	Internal Prediction (%)	External Prediction (%)	Efficiency (%)	Efficiency Forced Model (%)	Total Rate (%)
ALS (T0) vs. Controls	100	83.33	100	100	100	100

**Table 3:** The results of discriminant and modeling powers for each category of ten selected IR variables.

Spectra Variable	Discriminant Power	Modeling Power	
		Category HC	Category ALS (T0)
1234	9.07	0.94	0.99
1475	8.46	0.93	0.96
1310.5	7.72	0.92	0.92
1033.5	7.30	0.90	0.87
1400.5	7.22	0.90	0.85
1355	7.15	0.92	0.88
1180	6.99	0.93	0.88
1335.5	6.41	0.96	0.86
1449.5	6.32	0.98	0.92
1185	6.23	0.99	0.92

**Table 4:** Results of SELECT-LDA classification performance on 10 IR-selected spectral variables to discriminate between ALS T0, ALS T6, and ON.

Group	Classification (%)	Prediction (%)	External Prediction (%)	Total Rate (%)
ALS (T0)	100	86.67	100	95.55
ALS (T6)	96.31	81.82	100	92.71
ON	100	100	100	100
Total rate	98.73	87.50	100	95.41

## Data Availability

The original contributions presented in the study are included in the article/Supplementary Materials, further inquiries can be directed to the corresponding author/s.

## Supplementary Materials

The following supporting information can be downloaded at: https://www.mdpi.com/article/10.3390/bios14110526/s1, Figure S1: Raw spectra; Figure S2: Pre-processed spectra; Figure S3: The Cooman’s plots with 95% confidence level to define the class space and the unweighted augmented distance.; Figure S4: The permutation analysis between one predictive (p1) and one orthogonal (o1) components produced the observed and cross-validated R2X (in blue), R2Y (in pink), and Q2 (in lilac) coefficients for OPLS-DA model to differentiate ALS (T0) and control groups, Figure S5: PLS-DA discriminative analysis between ALS T0, ALS (T6) and ON group; Table S1: Comparative table of the 10 most important selected spectral variables by SELECT algorithm and by VIP score in each classification problem.; Table S2: Prediction matrix of true assigned to class category among ALS (T0), ALS (T6) and ON samples.
