# Supplementary material for: Comparative Blood Profiling Based on ATR-FTIR Spectroscopy and Chemometrics for Differential Diagnosis of Patients with Amyotrophic Lateral Sclerosis—Pilot Study
Source: Biosensors (Basel). 2024 Oct 30;14(11):526. doi: 10.3390/bios14110526 (PMC11591577; doi:10.3390/bios14110526)

Supplementary material

Figure S1: Raw spectra.

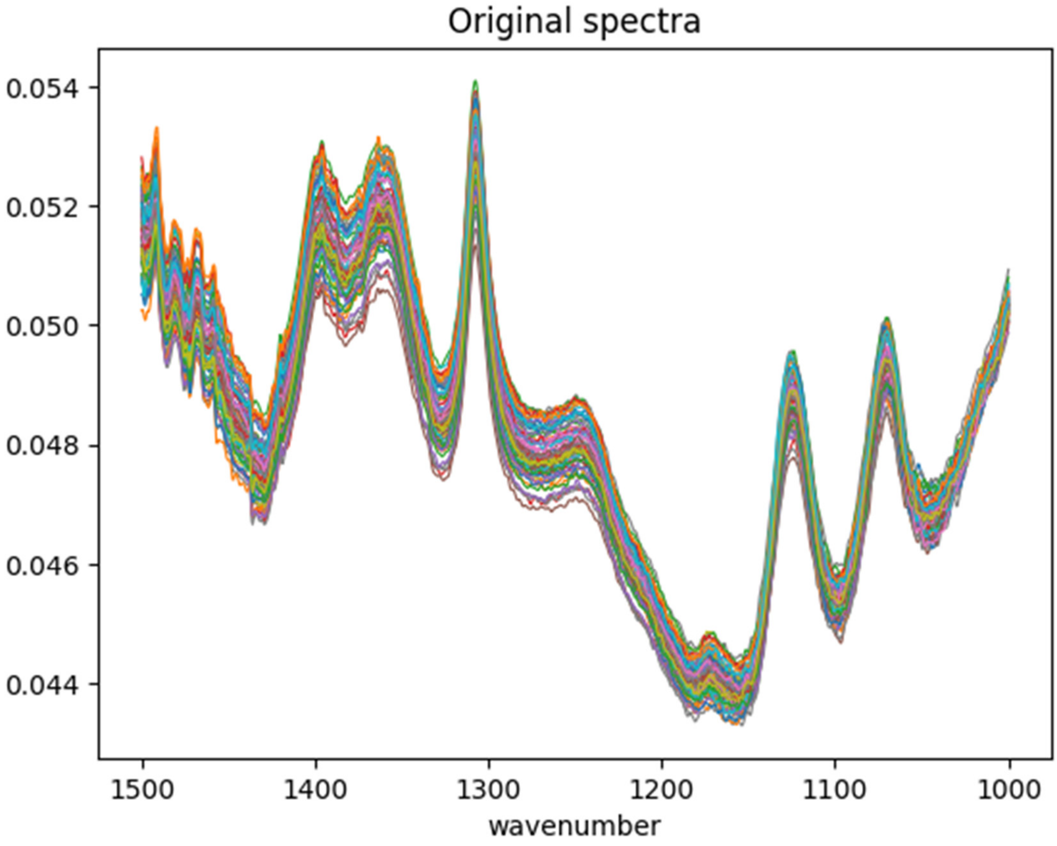

Figure S2: Pre-processed spectra.

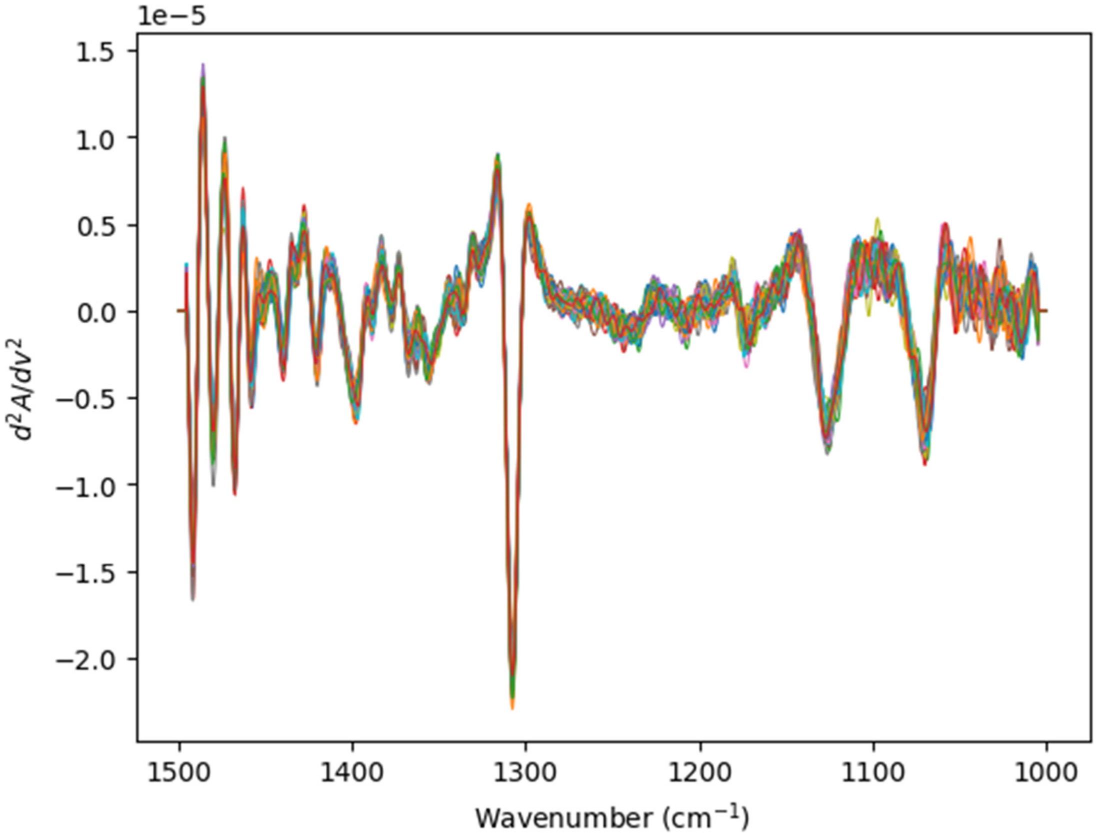

Figure S3: The Cooman's plots with 95% confidence level to define the class space and the unweighted augmented distance. This diagram displays the results obtained by applying SIMCA class modelling to patient categories ALS (T0) (■) and controls (■) within the included test set (O). The red dashed line indicates equal class distance.

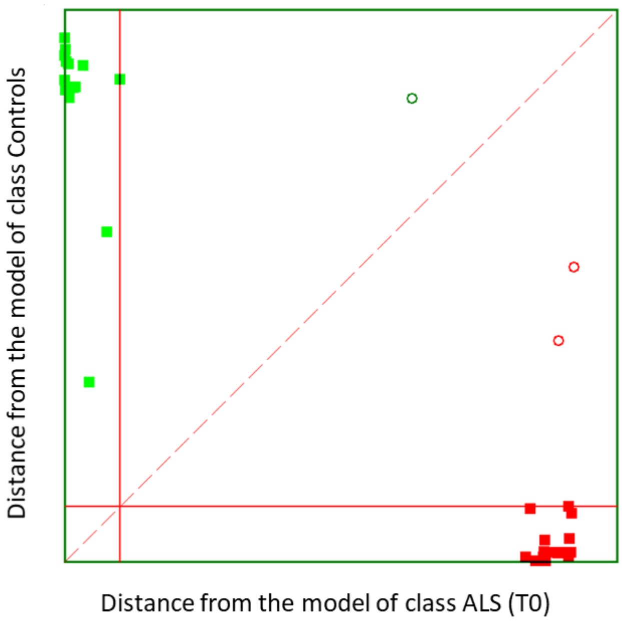

Figure S4: The permutation analysis between one predictive (p1) and one orthogonal (o1) components produced the observed and cross-validated R2X (in blue), R2Y (in pink), and Q2 (in lilac) coefficients for OPLS-DA model to differentiate ALS (T0) and control groups.

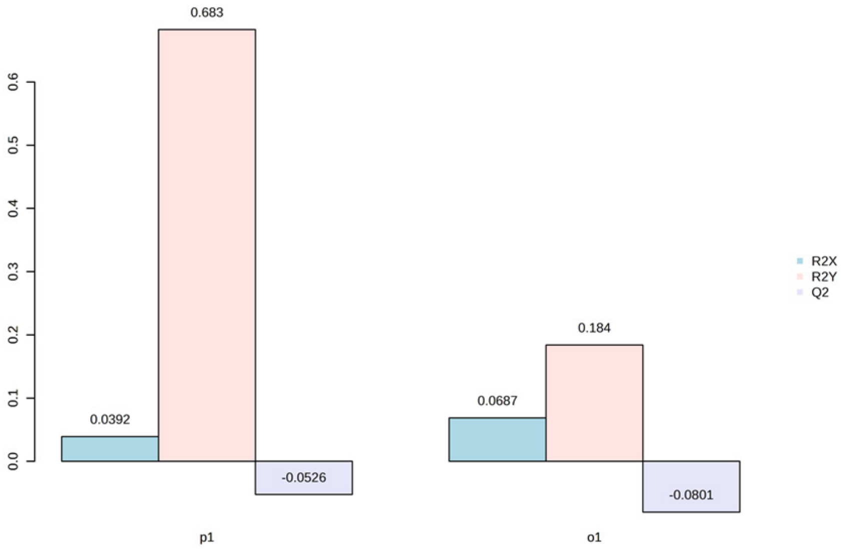

Table S1: Comparative table of the 10 most important selected spectral variables by SELECT algorithm and by VIP score in each classification problem. (*) indicates those spectra variables selected in by different classification strategies but for the same classification problem; [] indicate the band that was selected by the same classification approach but for different classification problems.

**ALS (T0) vs Controls**		**ALS (T0) vs ALS (T6) vs ON**	
SELECT	VIP of OPLS-DA model	SELECT	VIP of PLS-DA model
1180	1205	1045	***1304**
1185	1264	1106	1120
1234	1269	1135.5	1121
1355	1335	1150.5	1303
1475	1204.5	1231.5	1305
1033.5	1205.5	***1304**	1119.5
1310.5	1263.5	1319	1120.5
***1335.5**	1264.5	1341.5	1303.5
1400.5	1269.5	1393.5	1304.5
**[1449.5]**	***1335.5**	**[1449.5]**	1305.5

Table S2: Prediction matrix of true assigned to class category among ALS (T0), ALS (T6) and ON samples

**Class**	*ALS (T0)*	*ALS (T6)*	*ON*
ALS (T0)	13	1	1
ALS (T6)	2	9	0
ON	0	0	6
**% Correct predictions**	86.67	81.82	100.00

Figure S5: PLS-DA discriminative analysis between ALS T0 (red T2 Hotelling's ellipses with a 95% confidence level), ALS (T6) (green T2 Hotelling's ellipses with a 95% confidence level) and ON group (blue T2 Hotelling's ellipses with a 95% confidence level).

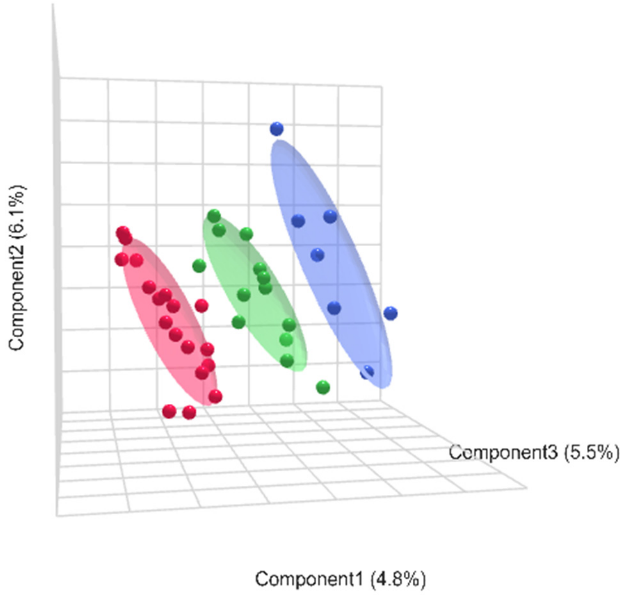

Supplement: Supplementary file 1 [file biosensors-14-00526-s001.zip › biosensors-3201974-supplementary.pdf]
